# Regulating Solvent‐Separated Ion Pairs to Control Polysulfide Redox for Fast and Stable Room‐Temperature Na‐S Batteries

**DOI:** 10.1002/advs.202521752

**Published:** 2026-03-02

**Authors:** Xiang‐Long Huang, Xue Li, Hengjia Shao, Xinran Gao, Zhengzhao Huang, Lingfei Zhao, Long Yao, Kunjie Zhu, Fu‐Sheng Ke, Hua‐Kun Liu, Wei‐Hong Lai, Yun‐Xiao Wang

**Affiliations:** ^1^ Institute of Energy Materials Science University of Shanghai for Science and Technology Shanghai P. R. China; ^2^ Guangdong Provincial Key Laboratory of Service Safety for New Energy Materials College of Materials Science and Engineering Shenzhen University Shenzhen P. R. China; ^3^ College of Chemistry and Molecular Sciences Wuhan University Wuhan P. R. China; ^4^ Institute For Superconducting and Electronic Materials Australian Institute of Innovative Materials Innovation Campus University of Wollongong Wollongong Australia; ^5^ Laboratory of Advanced Materials Shanghai Key Laboratory of Molecular Catalysis and Innovative Materials School of Chemistry and Materials Fudan University Shanghai P. R. China

**Keywords:** interface chemistry, local high‐concentration electrolyte, Na‐S batteries, redox kinetics, solvent‐separated ion pairs

## Abstract

The solvent‐separated ion pairs in local high‐concentration electrolytes (LHCE) have a profound influence on rechargeable sulfur‐based batteries yet are always ignored. Herein, tailored solvent‐separated ion pairs are engineered by tuning the diluent ratio in LHCE to boost sulfur redox kinetics in room‐temperature sodium‐sulfur (Na‐S) batteries. The tailored solvent‐separated ion pairs allow the sparingly dissolved polysulfides to enhance localized solid‐liquid‐solid transformation on reactive interfaces, so the LHCE decorated with the solvent‐separated ion pairs preserves the global quasi‐solid‐state mechanism of sulfur while modulating its intrinsic sulfur redox kinetics. Consequently, Na‐S batteries achieve excellent cyclability (fading rate of 0.017% per cycle over 2400 cycles at 1.0C) and outstanding rate performance (564 mA h g^−^^1^ at 2.0C), and the pouch cells can deliver an ultrahigh capacity of 912 mA h g^−^^1^ at 0.1C. This work highlights the critical role of SSIPs in enabling fast and stable sulfur chemistry for durable and fast‐charging Na‐S batteries.

## Graphical abstract

A local high‐concentration electrolyte with tailored solvent‐separated ion pairs is pioneered to design for room‐temperature sodium‐sulfur batteries. This electrolyte allows sparingly dissolved polysulfides and localizes them onto reactive interfaces for liquid‐solid conversion. It is able to boost the global quasi‐solid‐state sulfur redox kinetics through enhanced localized solid‐liquid‐solid conversion. Such a tailored electrolyte can realize the trade‐off between electrochemical stability and kinetics in room‐temperature sodium‐sulfur batteries.

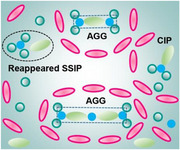

## 1 Introduction

**FIGURE 1:**
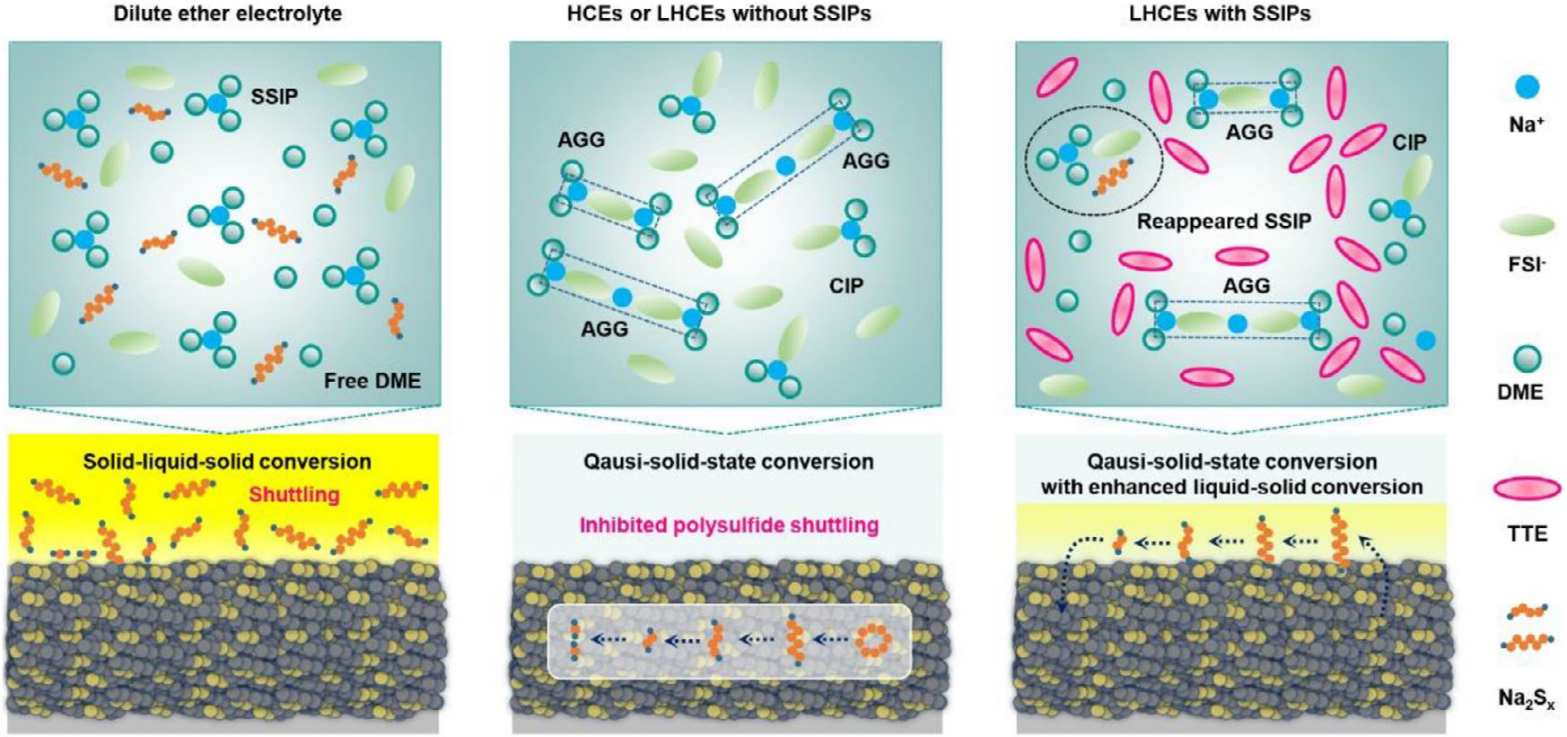
Schematic illustrations of different electrolyte solvation structures and corresponding sulfur redox mechanisms for Na‐S batteries.

Room‐temperature sodium‐sulfur (Na‐S) batteries have emerged as an exceptionally promising electrochemical energy storage technology in recent years [1, 2]. Their appeal stems from the unique two‐electron redox chemistry between sodium and sulfur, which confers an ultrahigh theoretical specific capacity of 1675 mA h g^−^^1^ and a high theoretical energy density of 1274 W h kg^−1^. Furthermore, they leverage abundant natural resources and low‐cost raw materials and offer significantly enhanced safety compared to their high‐temperature counterparts. In conventional Na‐S batteries utilizing strongly solvating electrolytes, such as dilute ether‐based formulations, sulfur conversion typically follows a solid‐liquid‐solid reaction pathway [3, 4]. This process electrochemically generates a large number of polysulfide intermediates during cycling. In such dilute electrolytes, the solvent‐separated ion pairs (SSIPs) dominated solvation structure permits a large number of polysulfide intermediates to dissolve and migrate between electrodes (Figure 1a), a phenomenon known as the shuttle effect of polysulfides. While this dissolution‐reprecipitation mechanism exhibits rapid intrinsic kinetics and improved active material utilization in Na‐S batteries, the severe shuttle effect may even cause the overcharging issue at the initial cycle (Figure S1) and thus the complete battery failure. In other words, the shuttle effect is a key factor to cause electrochemical instability in Na‐S batteries.

A reliable solution to the electrochemical instability in Na‐S batteries is to increase sodium salt concentration to form high‐concentration electrolytes (HCEs), strengthens coordination between sodium ions and free ether solvents, compels the solvation structure transformations from SSIPs to contact ion pairs (CIPs) and aggregate solvates (AGGs), and thus drastically reduce polysulfide solubility [5, 6]. The HCEs are able to trigger a unique quasi‐solid‐state conversion pathway (Figure 1b) while promote the generation of anion‐derived inorganic‐rich solid‐electrolyte interphase (SEI) layers for stabilized sodium anodes, enabling greatly enhanced electrochemical stability in Na‐S batteries. Nevertheless, their typical disadvantages in low ionic conductivity, ultrahigh viscosity, and poor wettability give rise to sluggish redox kinetics and thus inferior rate capabilities in Na‐S batteries. It is a praised method to incorporate diluents into HCEs and thereby design local high‐concentration electrolytes (LHCEs), since it can maintain the original solvation structures of HCEs, reduce electrolyte viscosity, reasonably elevate ionic conductivity, and enhance the electrolyte wettability. Despite successfully preserving the solvation structures of the corresponding HCE counterparts, most of LHCEs fail to alter the sulfur conversion pathway or significantly regulate reaction kinetics [7, 8, 9]. Accordingly, Na‐S batteries with the LHCEs still suffer from sluggish redox kinetics and inadequate rate performance, as evidenced by many studies [7, 8, 9, 10, 11]. Thus, it is still much challenging to approach the trade‐off between electrochemical stability and redox kinetics in Na‐S batteries featuring a quasi‐solid‐state conversion mechanism.

In this contribution, we pioneer to propose the reconstruction of SSIP structures in weakly solvating LHCEs in an attempt to break through the kinetics limitation for Na‐S batteries. A tailored diluent proportion is discovered to induce the reappearance of SSIPs in the LHCE, as confirmed by Raman spectra and small‐angle X‐ray scattering (SAXS). The reconstructed SSIPs allow the sparingly dissolved polysulfides for liquid‐solid conversion on reactive interfaces (Figure 1c), and the LHCE with the reconstructed SSIPs still prevents from the leakage and shuttling of polysulfides in the bulk electrolyte, due to its unaltered total weak solvation ability for polysulfide intermediates. The liquid‐solid conversion that is localized at the reactive interfaces can facilitate quasi‐solid‐state sulfur conversion kinetics in Na‐S batteries. Therefore, Na‐S batteries obtain superior rate performance with a high capacity of 564 mA h g^−1^ at 2.0C as well as an ultralow capacity fading rate of 0.017% per cycle over 2400 cycles at 1.0C. Furthermore, a high capacity of 912 mA h g^−1^ at 0.1C can be also approached in the pouch Na‐S cell employing the optimized LHCE.

## 2 Results and Discussion

### 2.1 Understanding the Reconstruction Mechanism of SSIPs

**FIGURE 2:**
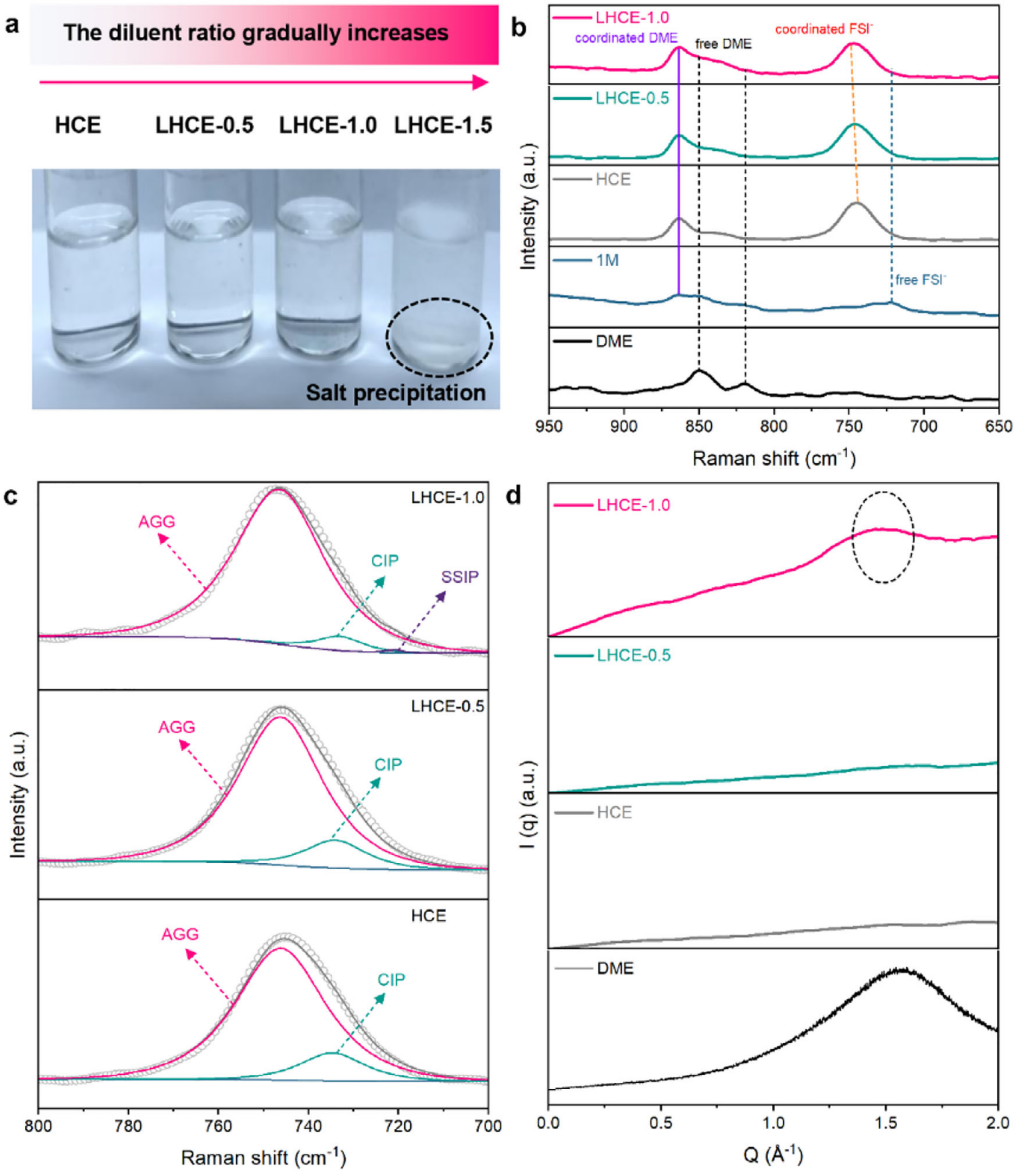
(a) Optical photographs of LHCEs with different diluent proportions. (b,c) Raman spectra of different electrolyte solutions. (d) 1D SAXS curves of DME solvent, HCE, LHCE0.5, and LHCE‐1.0.

For convenient analysis and in‐depth understanding, we selected a classic and representative electrolyte formula as the basis for designing the target electrolyte. Organic salt sodium bis(fluorosulfonyl)imide (NaFSI) was dissolved in strongly solvating main solvent 1, 2‐Dimethoxyethane (DME) at a molecular molar ratio of 1:1.2 to form the based HCE. Subsequently, a non‐solvating diluent 1,1,2,2‐tetrafluoroethyl 2,2,3,3‐tetrafluoropropyl ether (TTE) was added into this concentrated system at molar ratios of 0.5, 1.0, and 1.5, respectively, to produce various LHCEs (marked as LHCE‐0.5, LHCE‐1.0, and LHCE‐1.5). Figure 2a clearly shows the optical images of these as‐designed electrolytes. When the molar ratio of TTE reaches 1.5, part of the sodium salt recrystallizes and reprecipitates from the electrolyte solution. This fully indicates that the original solvation structure in the HCE may no longer be adequately maintained and also implies that the uncoordinated free DME solvent molecules are re‐generated within the corresponding LHCE environment. Thus, the HCE, LHCE‐0.5, and LHCE‐1.0 will be used to perform relevant analysis.

It has been widely recognized that the dramatically increased salt ratio promotes the transition of SSIPs into CIPs and AGGs in electrolytes [5, 12]. Accordingly, it can be reasonably inferred from the aforesaid salt reprecipitation phenomenon that the ever‐increasing diluent ratio promotes the gradual structure transition of LHCEs toward the direction of SSIPs reappearance while retaining the original coordination structures of HCEs and that the solvation structures of LHCEs are completely destroyed to pose the salt reprecipitation and electrolyte function failure when the incorporation ratio of diluent exceeds a certain threshold value. It must be noted that the salt reprecipitation in the LHCE cause the left electrolyte environment to resemble the common dilute ether‐based electrolytes and lose the function of diminishing polysulfide dissolution.

To verify the aforesaid estimation, Raman spectra of various electrolyte solutions were acquired to detect their changes in solvation structures (Figure 2b). As the salt concentration in DME solvent is increased to form the HCE, the Raman peak attributed to coordinated DME get obviously intensified and those peaks ascribed to free DME molecules completely vanish. Meanwhile, Raman peak from free FSI^−^ in 1 m dilute electrolyte also disappears with the increased salt concentration, and a strong peak ascribed to coordinated FSI^−^ occurs. After further incorporating different ratios of TTE diluent into the HCE, Raman spectra of the resulting LHCEs still exhibit strong peaks ascribed to coordinated DME and FSI^−^. Such changes indicate that the increased salt concentration promotes the coordination of sodium ions with DME and FSI^−^ to form strongly coordinated CIPs and AGGs as well as diminish free DME and FSI^−^. The peak ascribed to coordinated FSI^−^ undergoes an obvious blue shift with the increased TTE ratio, indicating the ever‐increased AGG proportion in electrolyte. In Figure 2c, the HCE and LHCE‐0.5 showcase the identical solvation structures, where there exist only CIPs and AGGs. However, for the LHCE‐1.0, the peak of CIPs is significantly reduced, the peak of AGGs is enhanced, and an extremely weak peak ascribed to SSIPs happens. This reveals that the enlarged TTE ratio promotes the CIP structure transformation toward greatly increased AGGs and a trace of SSIPs, that is, a few of free DME molecules are re‐generated in the LHCE‐1.0. These changes can be quantitatively understood by examining the proportions of the different solvation structures. In the HCE, the AGG‐dominated structures account for 86.7%, while CIPs constitute 13.3%. For LHCE‐0.5, the AGGs increase to 87.9%, and CIPs decrease to 12.1%, confirming that the increased TTE diluent favors AGG formation. Notably, in LHCE‐1.0, the proportion of AGGs rises further to 95.0%, whereas CIPs decline to 4.6%, and SSIPs appear at 0.4%.

The advanced SAXS technique was further adopted to verify the presence of free DME solvent molecules in the tailored LHCE‐1.0 (Figure 2d). The pure DME solution displays a broad scattering peak centered around Q = 1.5 Å^−1^, manifesting the presence of periodically arranged molecular clusters in free DME solvent. When a high concentration of salt is added to the DME solvent to form the HCE, the scattering peak disappears, indicating the absence of free DME solvent molecules. With the addition of non‐solvating TTE molecules at a molar ratio of 0.5, the corresponding SAXS pattern still show no scattering peak ascribed to free DME solvents. This demonstrates that the resulting LHCE‐0.5 maintains the solvation structures of pristine HCE at this case, without the re‐generation of free DME solvents. As the TTE ratio is increased to 1.0, a weak broad peak ascribed to free DME solvents reappears in the pattern, indicating the re‐generation of free DME solvents in the resulting LHCE‐1.0. These results further confirm the conclusion obtained through Raman spectra.

Based on the aforementioned analysis, it can be concluded that through tuning the TTE ratio, the rationally engineered LHCE‐1.0 possesses trace reconstructed SSIPs under the premise of retaining the AGG‐dominant solvation structures. Such a tailored electrolyte is likely able to constitute profound influence on sulfur redox reactions in Na‐S batteries.

### 2.2 The Influence of SSIPs on Quasi‐Solid‐State Sulfur Conversion

**FIGURE 3:**
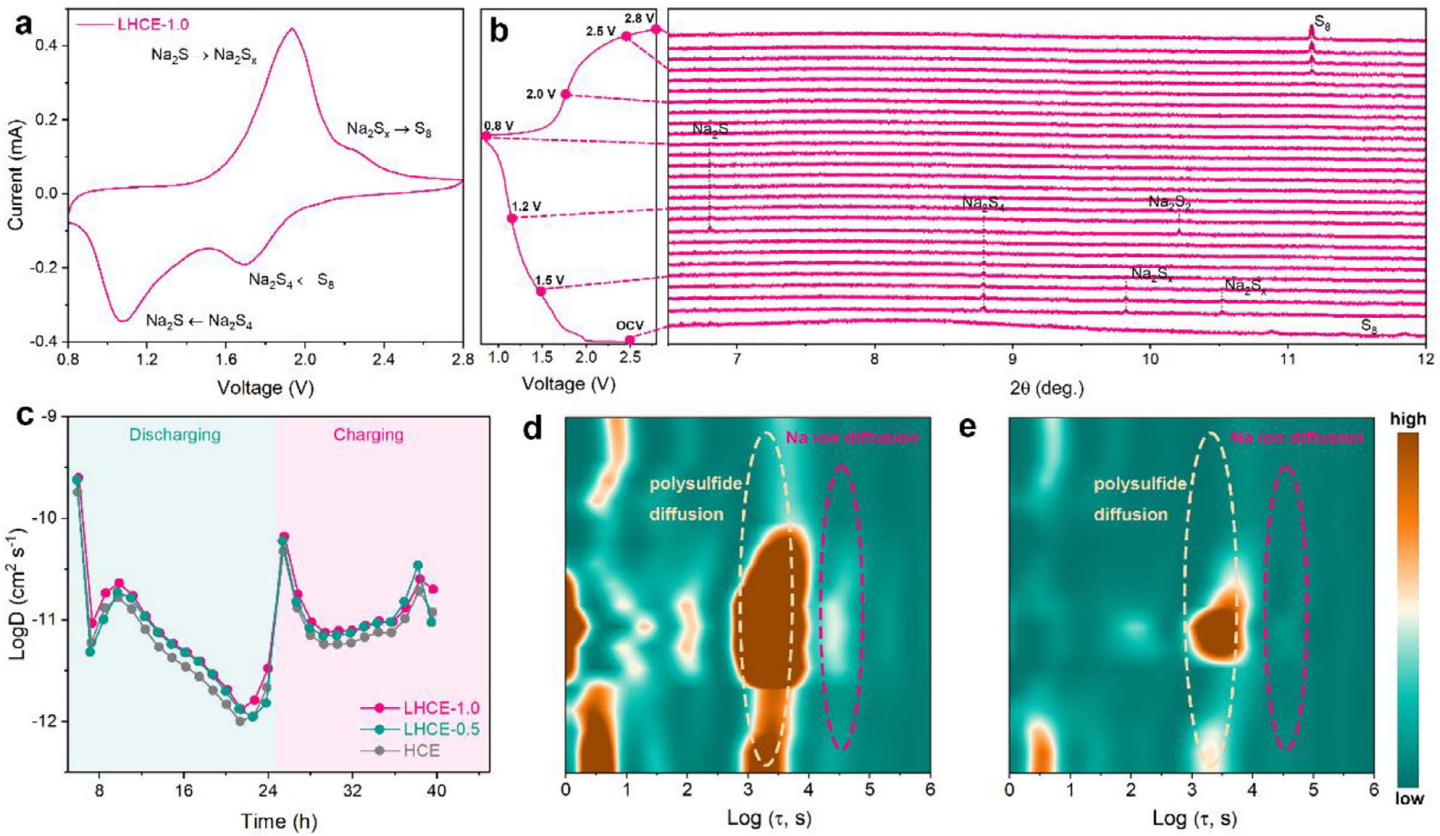
(a) Cyclic voltammetry (CV) curve of Na‐S batteries with the tailored LHCE‐1.0. (b) In situ synchrotron XRD patterns of Na‐S battery with the tailored LHCE‐1.0, accompanied with the corresponding discharge–charge profile. (c) Na^+^ diffusion coefficients obtained from GITT curves with different electrolytes during the charge and discharge process. (d,e) In situ DRT contour maps decoupled from in situ EIS Nyquist plots based on the HCE and LHCE‐1.0.

It needs to be urgently clarified how the emerging SSIPs in the LHCE‐1.0 influences the sulfur conversion mechanism. The reconstructed SSIPs in the LHCE‐1.0 allow sparingly dissolved polysulfides (i.e., forming liquid‐phase polysulfides), and the existing TTE molecules cannot impact this process according to the hybrid solvation theories [13, 14, 15]. Therefore, a conventional solid‐liquid‐liquid conversion pathway must exist in Na‐S batteries with such an electrolyte system. Meanwhile, the AGG‐dominant solvation structures in LHCE‐1.0 are generally maintained to prevent polysulfides from migrating toward the sodium anode and thus inhibit their shuttling behaviors, that is, sulfur is still compelled to undergo quasi‐solid‐state sulfur conversions as it does in conventional HCEs and LHCEs [16, 17, 18]. As a result, the induced solid‐liquid‐solid conversion process can only take place on the near reactive surfaces/interfaces of the cathode side, where quasi‐solid‐state sulfur conversion occurs. The CV curve in Figure 3a clearly presents two pairs of broad redox peaks. Two reduction peaks reflect the conversion from sulfur to Na_2_S_4_ and the conversion from Na_2_S_4_ to Na_2_S, respectively. Conversely, two oxidation peaks represent reversible conversion from Na_2_S to polysulfides and finally to sulfur. The discharge–charge profile of Na‐S batteries with the tailored LHCE‐1.0 in Figure S2 further confirms the conversion process, where the plateau voltages are highly consistent with those of redox peaks. This suggests that the sulfur conversion in Na‐S batteries with the tailored LHCE‐1.0 still globally follows the quasi‐solid‐state redox reactions. Therefore, the sulfur conversion mechanism of Na‐S batteries with the LHCE‐1.0 can be adequately defined as quasi‐solid‐state redox reactions with enhanced localized liquid‐solid conversion.

We employed in situ synchrotron X‐ray diffraction (XRD) to further reveal the phase change mechanism in such a unique sulfur redox reaction pathway (Figure 3b). At the open‐circuit voltage, two peaks indexed to pristine S_8_ (PDF#97‐041‐9593) are detected. Upon initiating the discharge operation, characteristic peaks corresponding to higher‐order polysulfides (Na_2_S_x_, 4 < x ≤ 8) and Na_2_S_4_ (PDF#97‐000‐2586) emerge immediately, indicating rapid conversion of sulfur into polysulfides. As the discharging continues to 1.5 V, the characteristic peaks of Na_2_S_x_ completely disappear, and only the peak assignable to Na_2_S_4_ is left. Full conversion of all polysulfides into Na_2_S_4_ suggests its role as a stable intermediate phase. Upon discharging to 1.2 V, characteristic peaks attributable to Na_2_S_2_ (PDF#97‐004‐3405) and Na_2_S (PDF#97‐009‐2771) start to emerge, confirming the transformation of Na_2_S_4_ into shorter‐chain sulfides. When the voltage further drops to 1.0 V, the characteristic peak of Na_2_S_2_ is no longer detectable and only the characteristic peak of Na_2_S remains, indicating that the reduction from Na_2_S_2_ to Na_2_S occurs primarily between 1.2 and 1.0 V. At the cut‐off voltage of 0.8 V, only the characteristic peak of Na_2_S is observed, confirming it as the final discharge product. Upon charging to 2.5 V, a strong characteristic peak corresponding to sulfur (PDF#97‐041‐2326) reemerges and its intensity increases continuously, manifesting the reformation of sulfur. This phase change behavior is corroborated by ex situ XPS analysis in Figure S3, where featuring peaks of Na_2_S_x_, Na_2_S_4_, Na_2_S_2_, and Na_2_S are successively observed at different discharge states, and peak signal of sulfur reappears after charging to 2.8 V.

Such a reaction mechanism is obviously different from the previous understanding of sulfur electrochemistry in conventional electrolyte systems. In conventional carbonate electrolyte, the reaction is dominated by a direct slow conversion from S_8_ to Na_2_S. The process lacks well‐defined plateaus corresponding to soluble long‐chain polysulfides (Na_2_S_x_, 4 ≤ x ≤ 8), indicating severely hindered kinetics and a probable solid‐solid transformation mechanism. This is consistent with literature reports where thick, resistive, and organic‐inorganic hybrid CEI layers formed by carbonates, blocking efficient ion/electron transport and preventing the dissolution‐reprecipitation process that is typically beneficial for full sulfur utilization. In a conventional ether electrolyte, sulfur redox reactions follow a solid‐liquid‐solid pathway. Such a reaction pathway mediated by natural liquid‐phase polysulfides can overcome the high energy barrier from solid sulfur to solid Na_2_S, but a large number of polysulfides is difficult to rapidly convert into Na_2_S, thus posing the severe shuttle effect (i.e., overcharging behaviors). In contrast, such unique quasi‐solid‐state sulfur redox reactions enhanced by surface‐localized liquid‐solid conversion are expected to realize faster reaction kinetics, higher reaction reversibility, and greatly boosted cycling stability in Na‐S batteries.

Next, the focus is transferred to the influence of the reconstructed SSIPs in the LHCE‐1.0 on sulfur redox reaction kinetics. The galvanostatic intermittent titration (GITT) profiles in Figure S4 reveal substantially lower overall polarization in Na‐S batteries with the LHCE‐1.0, as evidenced by a pronounced reduction in voltage hysteresis between charge and discharge steps in contrast to those of Na‐S batteries the HCE and LHCE‐0.5. Furthermore, Na‐S batteries with the LHCE‐1.0 manifest higher Na^+^ diffusion coefficients than those of the HCE and LHCE‐0.5 counterparts (Figure 3c). These results indicate superior reaction kinetics and reduced overall impedance when using the LHCE‐1.0. To evaluate sodium‐ion transport and polysulfide diffusion kinetics at different electrolytes, we recorded in situ electrochemical impedance spectroscopy (in situ EIS) of Na‐S batteries with different electrolytes during discharge–charge process and decoupled the interfacial dynamic process through employing the advanced technique of distribution of relaxation time (DRT). Figure S5 depicts the distribution of relaxation times (DRT) spectrum measured at open‐circuit voltage, where each characteristic peak corresponds to a distinct electrochemical process. The analysis reveals that the total cell polarization is predominantly governed by five resistance contributions: (i) concentration polarization, (ii) cathode‐electrolyte interphase (CEI) resistance, (iii) cathode charge transfer resistance, (iv) polysulfide diffusion impedance, and (v) electrolyte ion diffusion resistance [19, 20]. Figure S6 presents the DRT spectra acquired at varying voltages during dynamic monitoring, with corresponding DRT‐derived contour maps presented in Figure 3d,e and Figure S7. Particularly within the Warburg region (E region) reflecting dynamic variations in sodium‐ion diffusion resistance and polysulfide diffusion resistance, Na‐S batteries with the LHCE‐1.0 exhibit significantly lower sodium ion diffusion resistance and polysulfide diffusion resistance than corresponding counterparts. This pronounced contrast indicates the superior sodium‐ion conductivity in the LHCE‐1.0 as well as the higher solubility of polysulfides to render their easier interface diffusion, which jointly underlie superior sulfur redox reaction kinetics.

Generally, the LHCE modified with the reconstructed SSIPs can basically maintain the quasi‐solid‐state conversion mechanism in Na‐S batteries but these SSIPs allow the dissolution of relatively more polysulfides at reactive interfaces for localized enhanced liquid‐solid conversion. Such quasi‐solid‐state sulfur redox reactions with localized enhanced liquid‐solid conversion can realize faster reaction kinetics than those induced by common HCEs and LHCEs without SSIP structures.

### 2.3 The Correlation of SSIPs to Cathode Interface Chemistries

As evidenced by the past fundamental research [7, 9, 11], HCEs and LHCEs can induce thin and inorganic‐rich CEI layers on cathode surfaces through anion‐enhanced electrolyte chemistry to support the quasi‐solid‐state sulfur conversion. These anion‐derived CEI layers are usually believed to comprise NaF, Na_3_N, S‐F species, and a small number of organics, decomposed from organic sodium salt. It should be explored whether the reconstructed SSIPs in the LHCE constitute influence on cathode interface chemistries for Na‐S batteries, since the interfaces are closely related to sulfur redox reactions.

**FIGURE 4:**
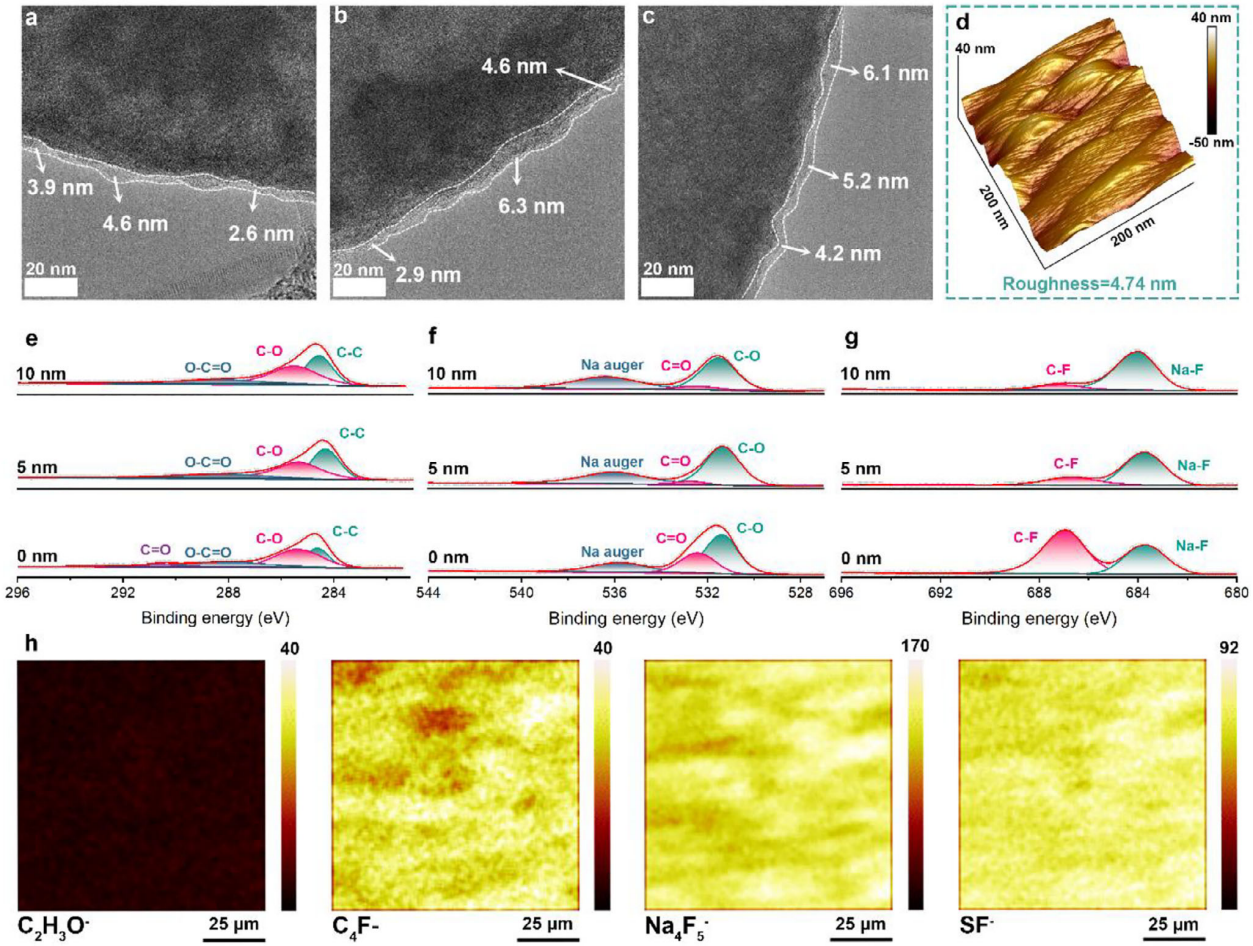
(a–c) HRTEM images of carbon/sulfur composites disassembled from Na‐S batteries with the LHCE‐1.0. (d) Atomic force microscopy (AFM) images of carbon/sulfur composites disassembled from Na‐S batteries with the LHCE‐1.0. (e–g) XPS spectra of the LHCE‐derived CEI layers in different etching depths: C 1s, O 1s, and F 1s. (h) 2D TOF‐SMIS images of sulfur cathode surfaces employing the LHCE‐1.0.

High‐resolution transmission electron microscopy (HRTEM) was further performed on multiple regions of the carbon/sulfur composites after cycling to observe the morphologies of CEI layers. As shown in Figure 4a–c, the LHCE decorated with the SSIPs can still build thin and thickness‐ homogeneous CEI layers with thicknesses ranging from approximately 3 to 6 nm. At the same time, the cathode surface from Na‐S batteries with the LHCE‐1.0 displays pronounced low average surface roughness of 4.74 nm (Figure 4d).

The chemical compositions of CEI layers were analyzed using X‐ray photoelectron spectroscopy (XPS). XPS survey spectrum in Figure S8 indicates the presence of Na, F, O, C, and S. As shown in Figure 4e,f, the C 1s spectrum reveals four distinct peaks at binding energies of 284.6 (C─C), 285.8 (C═O), 288.1 (O‐C═O), and 290.6 eV (C═O), and the O 1s spectrum exhibits characteristic peaks attributable to C═O and C─O bonds. This suggests the presence of organic species within the CEI layers [21, 22]. Although the organic species occupies a very small proportion in the entire CEI film, it can be detected that the peak intensity attributed to C═O bonds significantly decreases and even disappears with the increasing etching depths. This reveals mild spatial distribution heterogeneity of organic constituents in the CEI layer. F 1s spectrum reveals two peaks corresponding to NaF and C─F bonds, and the spatial distribution of NaF exhibits pronounced homogeneity with the changes in etching depths (Figure 4g). As the formation of CEI layers is dependent on the salt decomposition in the LHCE, S 2p spectrum exhibits a peak assignable to S‐F species (Figure S9), and N 1s spectrum confirms the presence of Na_3_N within the LHCE‐derived CEI layer (Figure S10). Besides, the spatial homogeneity of compositions is also embodied in the S‐F species, the characteristic peak signal of which can all be detected no matter at which etching depth (Figure S11). These results reveal the co‐existence of organic and inorganic components (NaF, Na_3_N, and S‐F species) in the CEI layers derived from the LHCE‐1.0 and the spatial homogeneity of the dominant inorganic components within the CEI layers.

Time‐of‐flight secondary ion mass spectrometry (TOF‐SIMS) was further employed to probe the chemical compositions and their distributions within the CEI layers (Figure 4h). Featuring ion fragments are clearly detected, including C_2_H_3_O^−^ (organic species), C_4_F^−^ (fluorocarbons), Na_4_F_5_^−^ (NaF), and SF^−^ (S‐F species). Figure S12 present the changes in signal intensity of these ion fragments with the increased sputtering depth, and Figure S13 further visually shows the 3D reconstructed images of TOF‐SIMS depth profiles for the CEI layers. It clearly shows the uniform distributions of these inorganic‐rich components within the CEI film.

The aforementioned analysis fully indicates the cathode interface chemistries induced by the SSIP‐decorated LHCE are highly consistent with those in common HCEs and LHCEs. This signifies that the tailored SSIP‐decorated LHCE can kinetically manipulate the quasi‐solid‐state sulfur redox reactions through enhanced localized liquid‐solid conversion but not alter the critical function of LHCEs in constructing inorganic‐dominant CEI layers.

### 2.4 SSIPs Boosted Electrochemical Performance

Considering the aforementioned unique properties and functions of the LHCE‐1.0 with the reconstructed SSIPs, it is expected to help tackle critical challenges in Na‐S batteries with common HCEs and LHCEs and realize fast and stable sulfur electrochemistry. Herein, the impact of the reconstructed SSIPs on electrochemical performance of Na‐S batteries were evaluated through employing the basic HCE without the SSIPs and the LHCE‐1.0 with the SSIPs.

**FIGURE 5:**
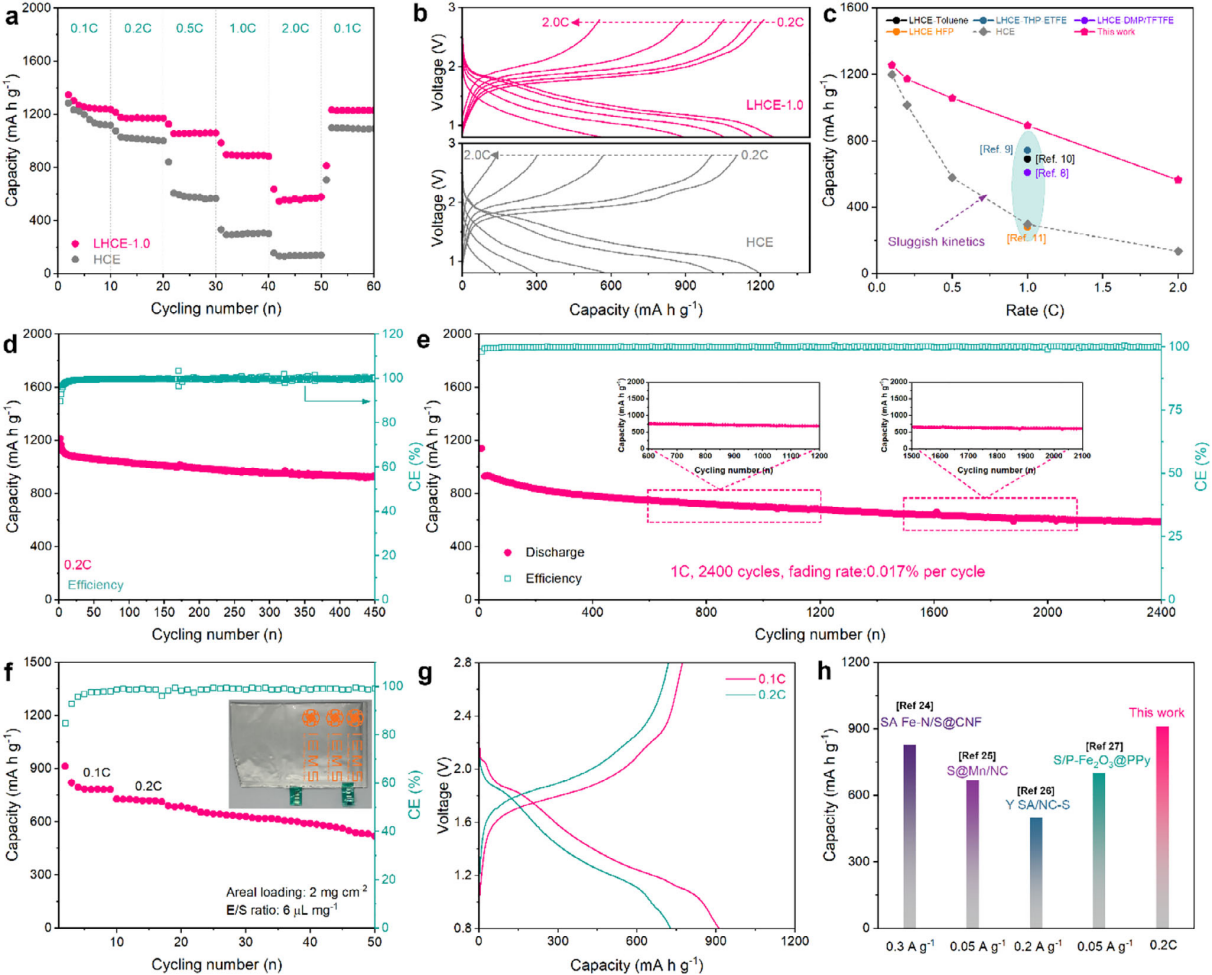
(a) Rate performance of Na‐S batteries with the LHCE‐1.0 and HCE at various current densities. (b) Discharge–charge profiles of Na‐S batteries with the LHCE‐1.0 and HCE. (c) Comparison of rate performance with previous literature [8, 9, 10, 11]. (d) Cyclability of Na‐S batteries with the LHCE‐1.0 at 0.2C. (e) Long‐term cycling performance at 1.0C. (f,g) Cycling performance and corresponding discharge–charge profiles of the Na‐S pouch cell. (h) Capacity comparison of this work with other reported Na‐S pouch cells [23, 24, 25, 26].

Na‐S batteries employing the LHCE‐1.0 deliver high capacities of 1254.8, 1171.8, 1056.4, 891.7, and 563.8 mA h g^−1^ at 0.1C, 0.2C, 0.5C, 1.0C, and 2.0C, respectively, which far outperform those with the HCE counterpart at the same conditions (Figure 5a). Na‐S batteries employing the LHCE‐1.0 can regain a high capacity of 1232.2 mA h g^−1^ after the C‐rate goes back to 0.1C and still retain an ultrahigh capacity of 1109 mA h g^−1^ even when continuously cycling to the 375th cycle (Figure S14). The corresponding discharge–charge profiles in Figure 5b reveals significantly reduced voltage polarization in Na‐S batteries with the LHCE‐1.0 compared to the HCE counterpart. Na‐S batteries with the LHCE‐1.0 can even keep well‐defined voltage plateaus even at 1.0C. Figure 5c further compares rate performance of this work with other Na‐S batteries employing LHCEs, fully demonstrating that the tailored solvation structures in the LHCE are able to effectively improve reaction kinetics and achieve high‐rate Na‐S batteries.

The cycling performance of Na‐S batteries employing the LHCE‐1.0 was further assessed at 0.2C to verify that the reconstructed SSIPs can maintain the critical role of LHCEs in promoting electrochemical stability in Na‐S batteries. As shown in Figure 5d, Na‐S batteries can preserve a high specific capacity of 925.8 mA h g^−1^ after cycling for 450 cycles. The corresponding discharge–charge profiles at different cycles show the identical voltage plateaus and no obvious capacity fading or voltage polarization (Figure S15). The superior cyclability at such a small rate fully validates that the tailored LHCE can boost sulfur redox reactions by promoting localized liquid‐solid conversion but not trigger the recidivation of the shuttle effect to pose rapid capacity fading. Furthermore, Na‐S batteries with the LHCE‐1.0 are pre‐cycled for 10 cycles at 0.2C and further cycled at a high rate of 1.0C to evaluate their long‐term cyclability. Impressively, Na‐S batteries exhibit outstanding durability in the ultralong cycling process exceeding six months (Figure 5e). To be specific, they demonstrate a high capacity of 993.5 mA h g^−1^ at 1.0C and still maintain 586 mA h g^−1^ over 2400 cycles, corresponding to an ultralow capacity fading rate of 0.017% per cycle. Figure S16 further highlights the excellent cyclability of Na‐S batteries with the LHCE‐1.0, where they manifest a far higher capacity retention than those with conventional electrolytes and carbon/sulfur composite cathodes. Na‐S batteries still harvest good cycling performance at 0.5C even if operating under a high areal sulfur loading (Figure S17).

To validate the practical applications of such a strategy of reinforcing LHCEs with the SSIPs, we further fabricated a Na‐S pouch cell with a size of 5 × 4 cm, a 100 µm‐ultrathin sodium belt, and a lean electrolyte/sulfur (E/S) ratio of 6 µm mg^−1^ to examine its cycling ability. The Na‐S pouch cell can produce a high reversible capacity of 912 mA h g^−1^ at 0.1C (Figure 5f), accompanied with a high energy density of 977.5 W h kg^−1^. In addition to that, the Na‐S pouch cell displays an almost identical discharge–charge profile at both 0.1C and 0.2C to those of coin cells (Figure 5g), signifying that the electrolyte‐tailored sulfur redox mechanism can be expanded to a larger scale. More importantly, the pouch cell exhibits no gas evolution and swelling even after undergoing a long‐term cycling process for 50 cycles (Figure S18), further indicating the robust interface stabilization function of the tailored LHCE. As illustrated in Figure 5h and Table S1, the as‐designed Na‐S pouch cell achieves remarkable performance positioning at the state‐of‐the‐art level of the field.

## 3 Conclusion

In summary, this work proposed an innovative concept of re‐generating SSIPs in LHCEs to overcome the limited kinetics and poor rate performance for Na‐S batteries. The Raman spectra and advanced SAXS scattering technique validated the successful formation of a tailored LHCE with reconstructed SSIPs through tuning the diluent ratios. In situ characterizations and electrochemical analysis revealed that the tailored electrolyte could not alter the general quasi‐solid‐state sulfur redox mechanism but manipulate sulfur reaction kinetics through enhancing localized liquid‐solid conversion. Besides, it was also found that the reconstructed SSIPs in the LHCE were not related to the cathode interface chemistries, that is, LHCEs with/without trace SSIPs exhibited the identical effects on the formation of CEI layers at sulfur cathode surfaces. Finally, the elaborate electrolyte enabled high capacity, superb rate performance, and outstanding cycling stability in Na‐S batteries. This work provides fundamental insights solvation chemistries of weakly solvating electrolytes, reveals their influence on sulfur redox reaction mechanisms, and establishes a new methodology for the design of high‐rate and durable room‐temperature sodium‐sulfur batteries.

## Conflicts of Interest

The authors declare no conflicts of interest.

## Supporting information




**Supporting File**: advs74679‐sup‐0001‐SuppMat.docx

## Data Availability

The data that support the findings of this study are available from the corresponding author upon reasonable request.

## References

[1] C. Ye , H. Li , Y. Chen , et al., “The Role of Electrocatalytic Materials for Developing Post‐Lithium Metal||Sulfur Batteries,” Nature Communications 15 (2024): 4797, 10.1038/s41467-024-49164-6. PMC11535197 38839870

[2] X. L. Huang , Y.‐X. Wang , S.‐L. Chou , S. X. Dou , and Z. M. Wang , “Materials Engineering for Adsorption and Catalysis in Room‐Temperature Na–S Batteries,” Energy & Environmental Science 14 (2021): 3757–3795, 10.1039/D1EE01349A.

[3] Q. Wu and Y. Qi , “Revealing Heterogeneous Electric Double Layer (EDL) Structures of Localized High‐Concentration Electrolytes (LHCEs) and their Impact on Solid–Electrolyte Interphase (SEI) Formation in Lithium Batteries,” Energy & Environmental Science 18 (2025): 3036–3046, 10.1039/D5EE00206K.

[4] Q. Zhang , T. Yang , and Z. Li , “Mechanism and Kinetics of Na_2_ S_x_ (x ≤ 2) Precipitation in Sodium‐Sulfur and Sodium/(Oxygen)‐Sulfur Batteries,” Journal of The Electrochemical Society 171 (2024): 010503, 10.1149/1945-7111/ad14cb.

[5] C. M. Efaw , Q. Wu , N. Gao , et al., “Localized High‐Concentration Electrolytes get more Localized Through Micelle‐Like Structures,” Nature Materials 22 (2023): 1531–1539, 10.1038/s41563-023-01700-3. 37932334

[6] J. Zheng , S. Chen , W. Zhao , J. Song , M. H. Engelhard , and J.‐G. Zhang , “Extremely Stable Sodium Metal Batteries Enabled by Localized High‐Concentration Electrolytes,” ACS Energy Letters 3 (2018): 315–321, 10.1021/acsenergylett.7b01213.

[7] J. He , A. Bhargav , W. Shin , and A. Manthiram , “Stable Dendrite‐Free Sodium–Sulfur Batteries Enabled by a Localized High‐Concentration Electrolyte,” Journal of the American Chemical Society 143 (2021): 20241–20248, 10.1021/jacs.1c08851. 34816711

[8] W. Yao , M.‐H. Pai , and A. Manthiram , “Inner–Outer Sheath Synergistic Shielding of Polysulfides in Asymmetric Solvent‐Based Electrolytes for Stable Sodium–Sulfur Batteries,” Journal of the American Chemical Society 147 (2025): 12061–12074, 10.1021/jacs.4c18374. 40135935

[9] W. Yao , M.‐H. Pai , and A. Manthiram , “Deciphering the Impact of Polysulfide Solvation Structure on Electrical Double Layer Chemistry in Sodium–Sulfur Batteries,” Angewandte Chemie International Edition 64 (2025): 202424547, 10.1002/anie.202424547. 40063424

[10] M.‐H. Pai , T. Lai , and A. Manthiram , “Sodium–Sulfur Cells with a Sulfurized Polyacrylonitrile Cathode and a Localized High Concentration Electrolyte With Toluene as a Nonfluorinated Diluent,” Advanced Functional Materials 34 (2024): 2407450, 10.1002/adfm.202407450.

[11] B. Jin , T. Lai , and A. Manthiram , “Locally Confined Polysulfide‐Reactive Electrolytes for Shuttle‐Free Sodium–Sulfur Batteries,” Journal of the American Chemical Society 147 (2025): 26414–26424, 10.1021/jacs.5c05389. 40692284

[12] D. Guo , J. Wang , Z. Cui , et al., “Low‐Temperature Sodium–Sulfur Batteries Enabled by Ionic Liquid in Localized High Concentration Electrolytes,” Advanced Functional Materials 34 (2024): 2409494, 10.1002/adfm.202409494.

[13] W. Chen , J.‐S. Park , C. Kwon , et al., “Hybrid Solvating Electrolytes for Practical Sodium‐Metal Batteries,” Joule 9 (2025): 101811, 10.1016/j.joule.2024.101811.

[14] L.‐P. Hou , X.‐Q. Zhang , N. Yao , et al., “An Encapsulating Lithium‐Polysulfide Electrolyte for Practical Lithium–Sulfur Batteries,” Chemistry (Weinheim An Der Bergstrasse, Germany) 8 (2022): 1083–1098, 10.1016/j.chempr.2021.12.023.

[15] J. Feng , T. Liu , H. Li , Y.‐S. Hu , H. Mao , and L. Suo , “Ultralight Electrolyte with Protective Encapsulation Solvation Structure Enables Hybrid Sulfur‐Based Primary Batteries Exceeding 660 Wh/kg,” Journal of the American Chemical Society 146 (2024): 3755–3763, 10.1021/jacs.3c10260. 38308639

[16] J. Zheng , G. Ji , X. Fan , et al., “High‐Fluorinated Electrolytes for Li–S Batteries,” Advanced Energy Materials 9 (2019): 1803774, 10.1002/aenm.201803774.

[17] K. Qin , S. Li , D. Yen , et al., “A Coordinated‐Anion‐Enriched Electrolyte for Lean‐Electrolyte Li–S Batteries,” ACS Energy Letters 9 (2024): 3869–3876, 10.1021/acsenergylett.4c00859.

[18] X. Chen , Y. Meng , D. Xiao , and L. Qin , “Empowering the Potassium–Sulfur Battery with Commendable Reaction Kinetics and Capacity Output by Localized High‐Concentration Electrolytes,” ACS Applied Materials & Interfaces 16 (2024): 24464–24472, 10.1021/acsami.3c19583. 38710103

[19] N. Song , J. Ma , Y. Liang , et al., “Phase and Orbital Engineering Effectuating Efficient Adsorption and Catalysis Toward High‐Energy Lithium−Sulfur Batteries,” Advanced Materials 37 (2025): 2420588, 10.1002/adma.202420588. 40072259

[20] G. Wu , T. Liu , Z. Lao , et al., “Optimizing s–p Orbital Overlap Between Sodium Polysulfides and Single‐Atom Indium Catalyst for Efficient Sulfur Redox Reaction,” Angewandte Chemie International Edition 64 (2025): 202422208, 10.1002/anie.202422208. 39676177

[21] S. Weng , Y. Liu , S. Lu , et al., “Unraveling the Multifunctional Mechanism of Fluoroethylene Carbonate in Enhancing High‐Performance Room‐Temperature Sodium‐Sulfur Batteries,” Angewandte Chemie International Edition 64 (2025): 202421602, 10.1002/anie.202421602. 39585782

[22] D. Liu , Z. Li , X. Li , et al., “Stable Room‐Temperature Sodium–Sulfur Batteries in Ether‐Based Electrolytes Enabled by the Fluoroethylene Carbonate Additive,” ACS Applied Materials & Interfaces 14 (2022): 6658–6666, 10.1021/acsami.1c21059. 35076203

[23] R. Bai , Q. Lin , X. Li , et al., “Toward Complete Transformation of Sodium Polysulfides by Regulating the Second‐Shell Coordinating Environment of Atomically Dispersed Fe,” Angewandte Chemie International Edition 62 (2023): 202218165, 10.1002/anie.202218165. 36918348

[24] H. Zhang , M. Wang , X.‐L. Huang , S. Lu , K. Lu , and X. Wu , “Atomic Manganese Manipulating Polysulfide Speciation Pathway for Room‐Temperature Na‐S Batteries,” CCS Chemistry 6 (2024): 2289–2304, 10.31635/ccschem.024.202303640.

[25] E. Zhang , X. Hu , L. Meng , et al., “Single‐Atom Yttrium Engineering Janus Electrode for Rechargeable Na–S Batteries,” Journal of the American Chemical Society 144 (2022): 18995–19007, 10.1021/jacs.2c07655. 36214519

[26] H. Zhang , B. Song , W. Zhang , et al., “Bidirectional Tandem Electrocatalysis Manipulated Sulfur Speciation Pathway for High‐Capacity and Stable Na‐S Battery,” Angewandte Chemie International Edition 62 (2023): 202217009, 10.1002/anie.202217009. 36494321

