# Supplementary material for: Regulating Solvent‐Separated Ion Pairs to Control Polysulfide Redox for Fast and Stable Room‐Temperature Na‐S Batteries
Source: Adv Sci (Weinh). 2026 Mar 2;13(27):e21752. doi: 10.1002/advs.202521752 (PMC13170214; doi:10.1002/advs.202521752)
Supplement: Supplementary file 1 — Supporting File: advs74679‐sup‐0001‐SuppMat.docx [file ADVS-13-e21752-s001.docx]

**Supporting Information**

**Experimental Section**

**Synthesis of carbon/sulfur composite**

A mixture of DMF and methanol (1:1, v/v) was prepared, and 1.3 g of 2-aminoterephthalic acid was added into it under stirring until complete dissolution. Then, 1 mL of titanium isopropoxide was added and vigorously stirred for one hour. The resulting suspension was transferred into a Teflon-lined stainless-steel autoclave and heated at 150°C for 6 hours. After cooling to room temperature, the product was collected by centrifugation, washed thoroughly with methanol at least three times, and dried overnight under vacuum at 70°C to obtain a yellow solid powder. This precursor was then annealed in a tube furnace under argon atmosphere with a heating rate of 5 °C min^-1^ up to 800°C and held for three hours. After cooling, black powder was obtained. The black powder was treated with 5% hydrofluoric acid (HF) solution overnight to remove metall-relevant species. The resulting porous carbon was collected and rinsed thoroughly. Subsequently, the as-obtained porous carbon was mixed with sulfur in a mass ratio of 1:2 and ground thoroughly. The mixture was heated at 155°C for twelve hours, followed by further heating at 300°C for three hours. After cooling, the final carbon/sulfur composite was obtained, the sulfur loading content of which is 55%.

**Synthesis of sulfur cathodes**

The carbon/sulfur composite, carbon nanotubes, and PVDF were mixed at a mass ratio of 8:1:1 in NMP solvent and grinded to a homogeneous slurry. The slurry was uniformly casted on a carbon-coated aluminum foil current collector and dried at 70°C overnight in a vacuum oven to volatilize NMP. The electrode was subsequently punched to form round disks with a diameter of 10mm. The areal mass loading of sulfur in each sulfur cathode is 1.1 mg cm^-2^. For preparing high-loading sulfur cathode, the abovementioned slurry was coated on carbon paper with a diameter of 10 mm.

**Synthesis of electrolytes**

Sodium bis(fluorosulfonyl)imide (NaFSI) was dried in a vacuum oven for 24 hours at 120℃. 1,2-Dimethoxyethane (DME) and 1,1,2,2-tetrafluoroethyl 2,2,3,3-tetrafluoropropyl ether (TTE) were dried with molecule sieves for 3 days. All the electrolytes were prepared inside an Ar-filled glove box with an oxygen/water content below 0.01 ppm. The dilute ether-based electrolyte is composed of 1M NaFSI in DME. The local high-concentration electrolyte was composed of DME, NaFSI, and TTE with a molar ratio of 1:1.2:0.5 and 1:1.2:1.

**Materials Characterizations**

The morphologies and nanostructures of all the samples were observed via field emission scanning electron microscope (ZEISS, GeminiSEM 300) and transition electron microscope (Tecnai G2 F20 S-TWIN). The elemental distributions were collected by energy dispersive spectrometry (EDS). The crystal structures and chemical states of all the samples were analyzed employing an X-ray diffractometer (XRD) with Cu K-alpha radiation and X-ray photoelectron spectra (XPS, Thermo Scientific ESCALAB 250Xi), respectively. The sulfur content in the carbon/sulfur composite was estimated using thermogravimetric analysis (TG 209, Germany). Time-of-Flight Secondary Ion Mass Spectrometry (TOF-SIMS) measurements were performed in the negative mode using a TESCAN MAIA3 instrument. An electrode was subjected to a pulsed 30 keV Ga^+^ (3nA) ion beam. The TOF-SIMS measurements were conducted across an electrode sample volume with a 100 µm x 100 µm sputtering area and approximately 2 µm sputtered depth.

**Electrochemical measurements**

These sulfur cathode disks were assembled into 2032-type coin cells in an argon-filled glove box with an oxygen/water content below 0.01 ppm. The diameter and thickness of the used sodium foils were 10 mm and 500 µm, respectively. The glass fiber disc (Whatman, GF/A) with a diameter of 16 mm served as separators. 45 μL of electrolyte was used in each coin cell. The galvanostatic discharge-charge tests were conducted on a LAND instrument testing system under a room temperature. The voltage window employed was 0.8-2.8 V. CV curves were recorded on electrochemical workstation (Biologic VMP-3).

**In-situ characterizations**

In-situ XRD was conducted at the Powder Diffraction beamline, Australian Synchrotron (ANSTO). The wavelength was 0.6885 Å using the NIST LaB6 660b standard reference material. In-situ EIS spectra were obtained through using the electrochemical working station (Biologic VMP-3).

**
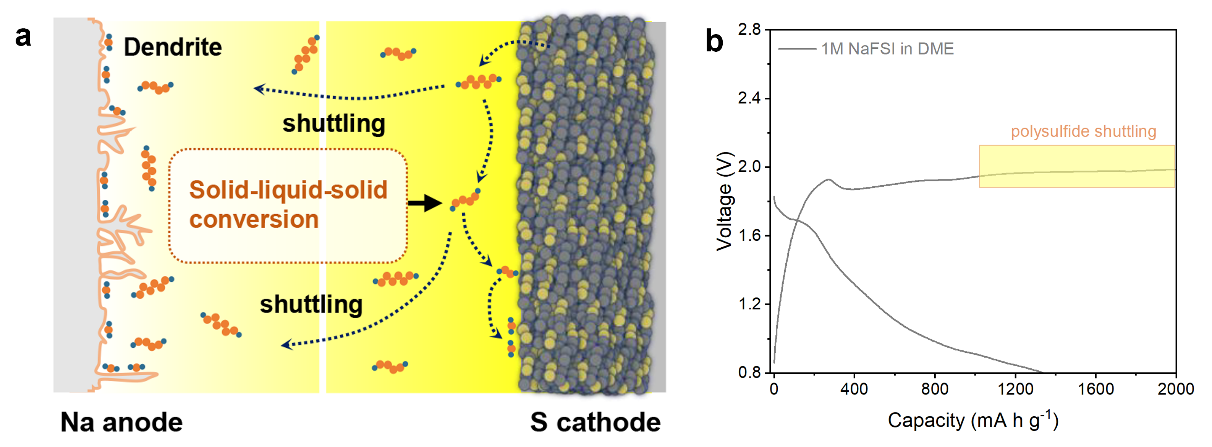
**

**Figure S1.** (a) Schematic illustration of solid-liquid-solid conversion mechanism in Na-S batteries. (b) Typical discharge-charge profile of Na-S batteries with the electrolyte of 1M NaFSI in DME.

**
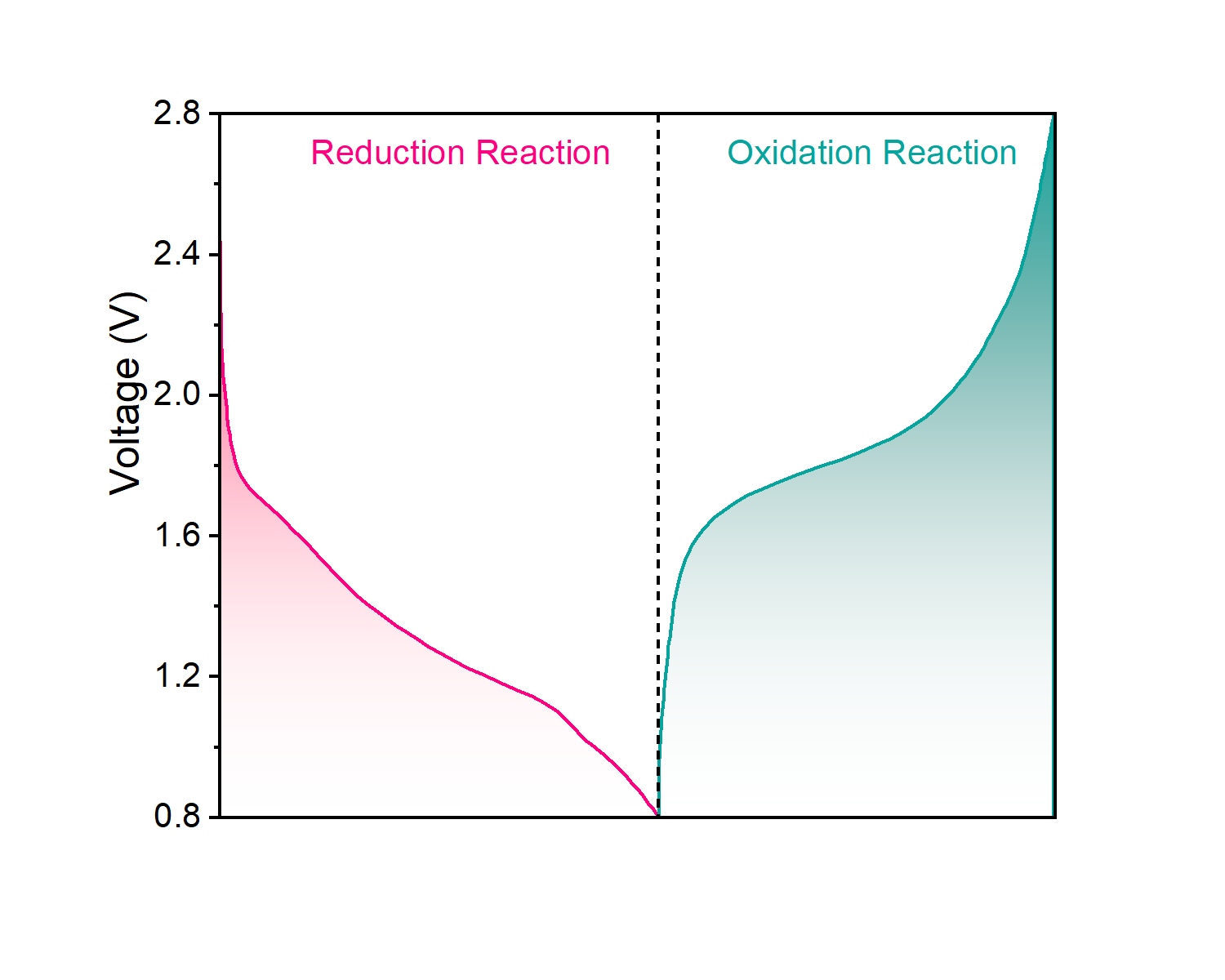
**

**Figure S2.** Discharge-charge profile of Na-S batteries with the tailored LHCE-1.0

**
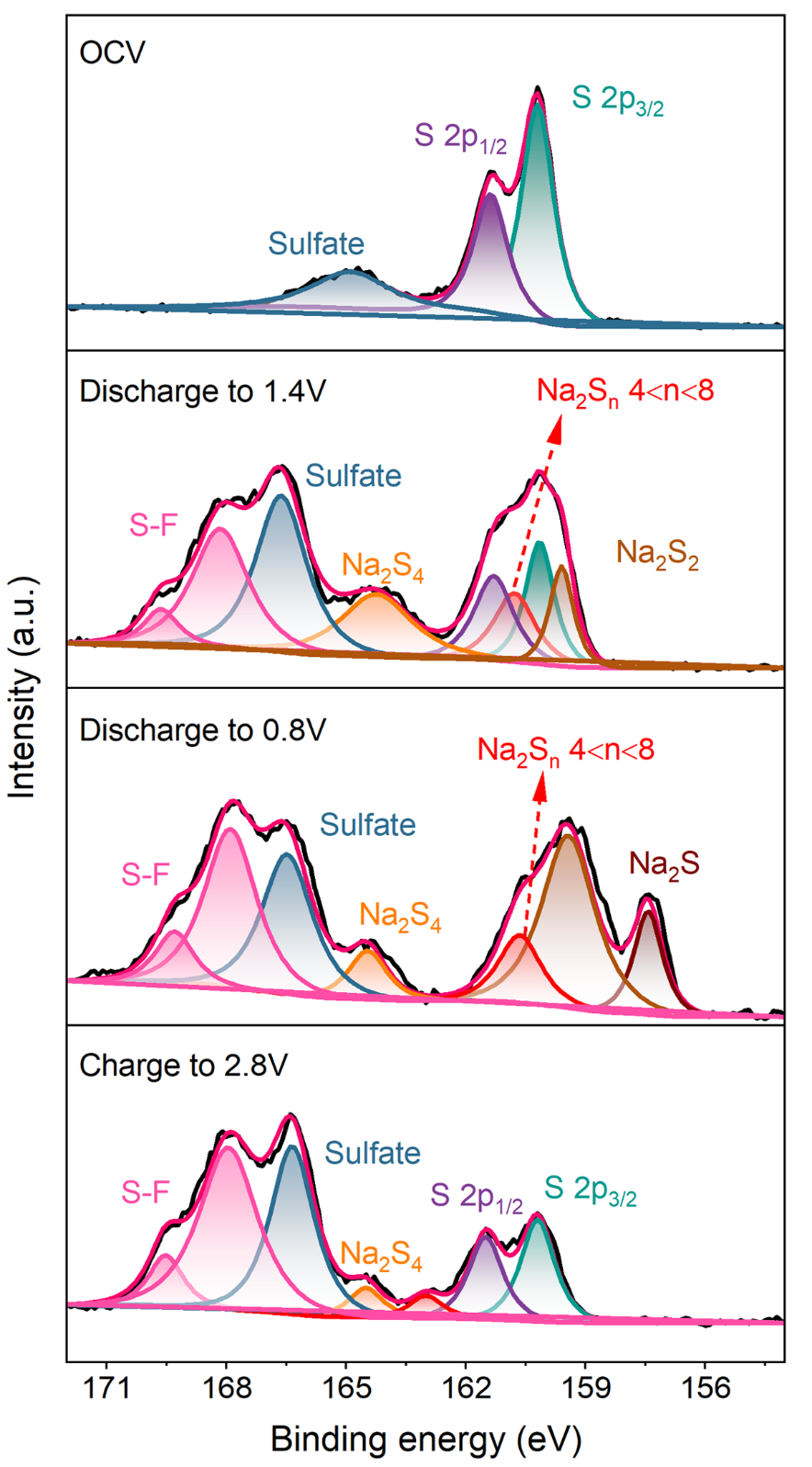
**

**Figure S3.** Ex-situ XPS spectra of S 2p for sulfur cathodes at different states.

**Figure S4.** GITT curves of Na-S batteries with different electrolytes.

**Figure S5.** Decoupled DRT peaks of initial EIS curves obtained by Na-S batteries with the LHCE-1.0.


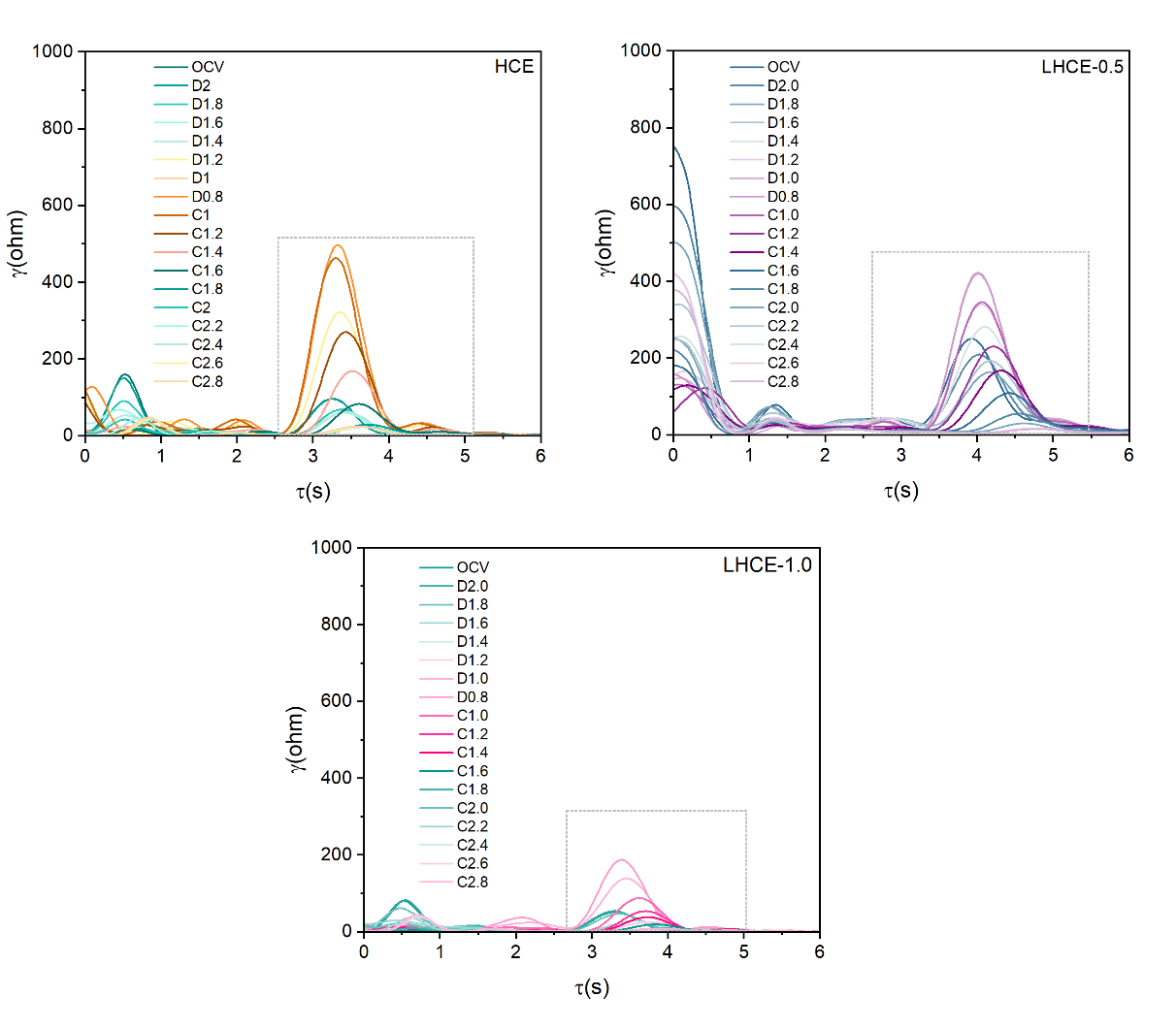


**Figure S6**. Decoupled DRT peaks of initial EIS curves obtained by Na-S batteries with different electrolytes.

**
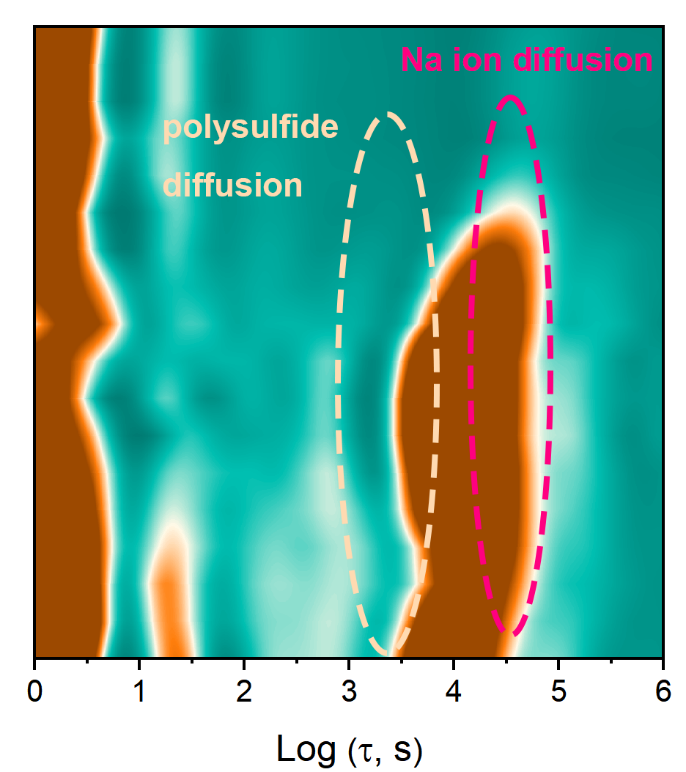
**

**Figure S7.** In-situ DRT contour maps decoupled from in-situ EIS Nyquist plots based on the HCE and LHCE-0.5.


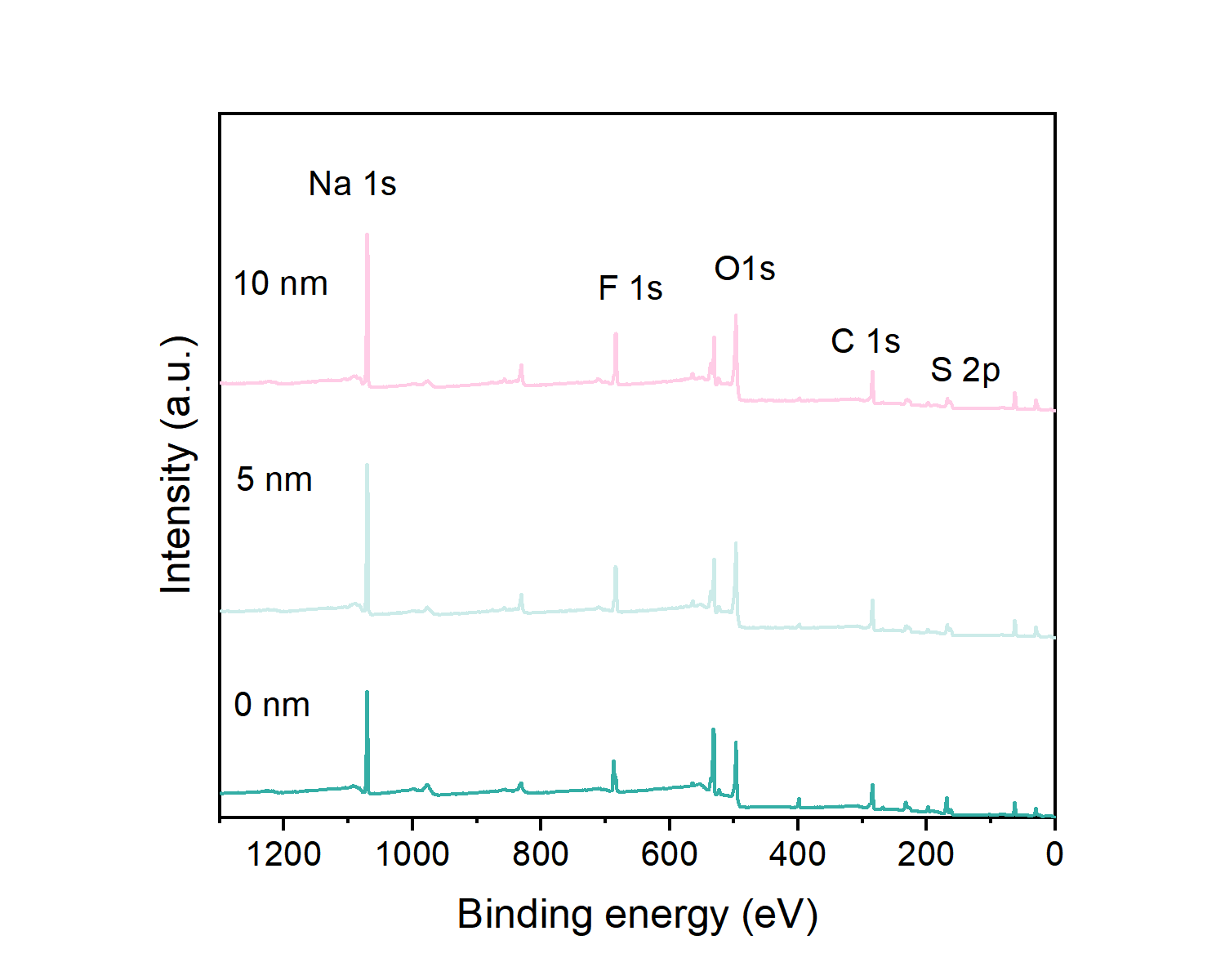


**Figure S8**. The XPS full spectra of sulfur cathodes after cycling at different depths.

**Figure S9.** S 2p spectrum of sulfur cathode after cycling in the LHCE.

**Figure S10.** N 1s spectrum of sulfur cathode after cycling in the LHCE.

**Figure S11.** S 2p spectrum of CEI film induced by the LHCE at different etching depths.


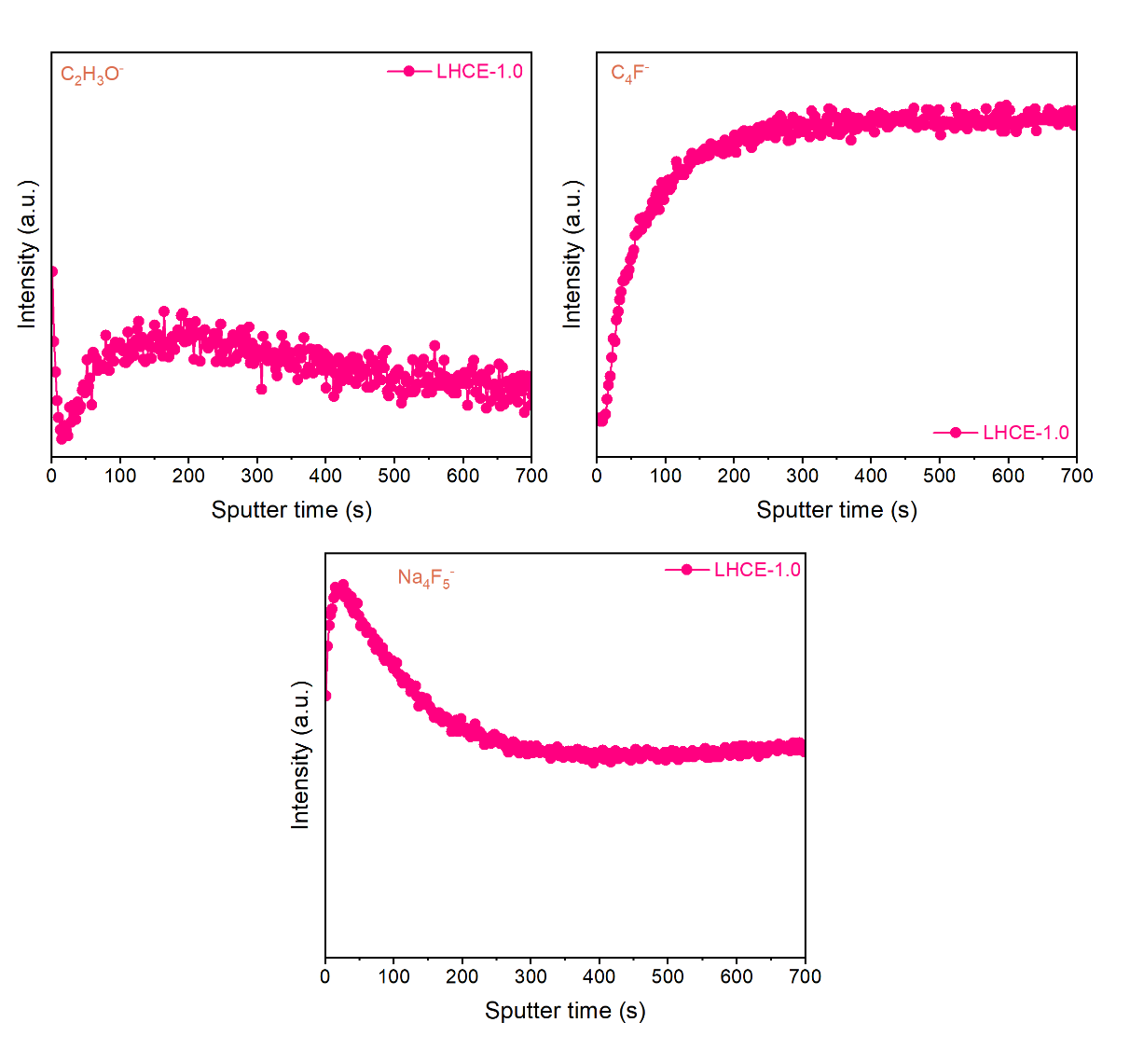


**Figure S12**. Depth profiles of different ion fragments for the cycled sulfur cathodes with the LHCE-1.0: C_2_H_3_O^-^, C_4_F^-^, andNa_4_F_5_^-^.

**
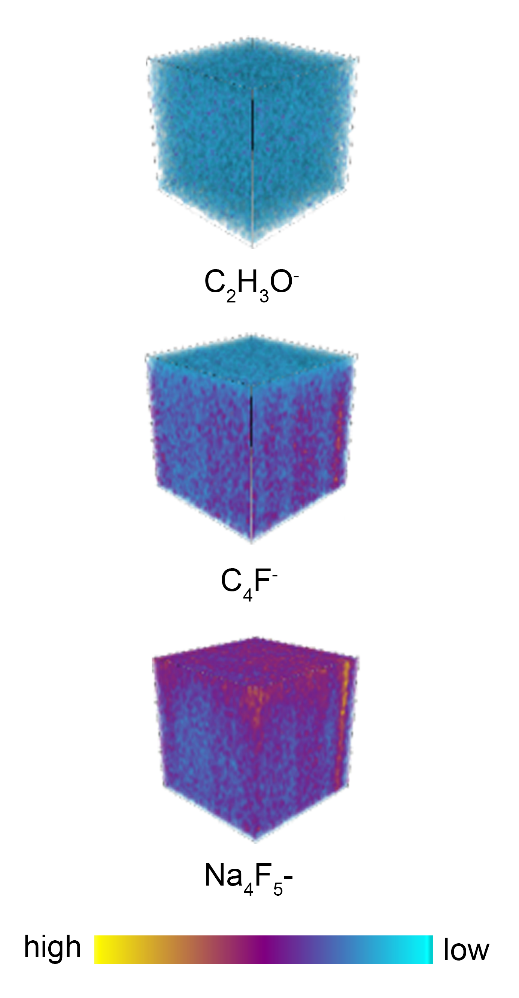
**

**Figure S13.** 3D reconstructed images of different ion fragments for sulfur cathodes with the LHCE-1.0 after the initial discharge.

**Figure S14.** Cycling stability of Na-S batteries with the LHCE after experiencing rate tests.

**
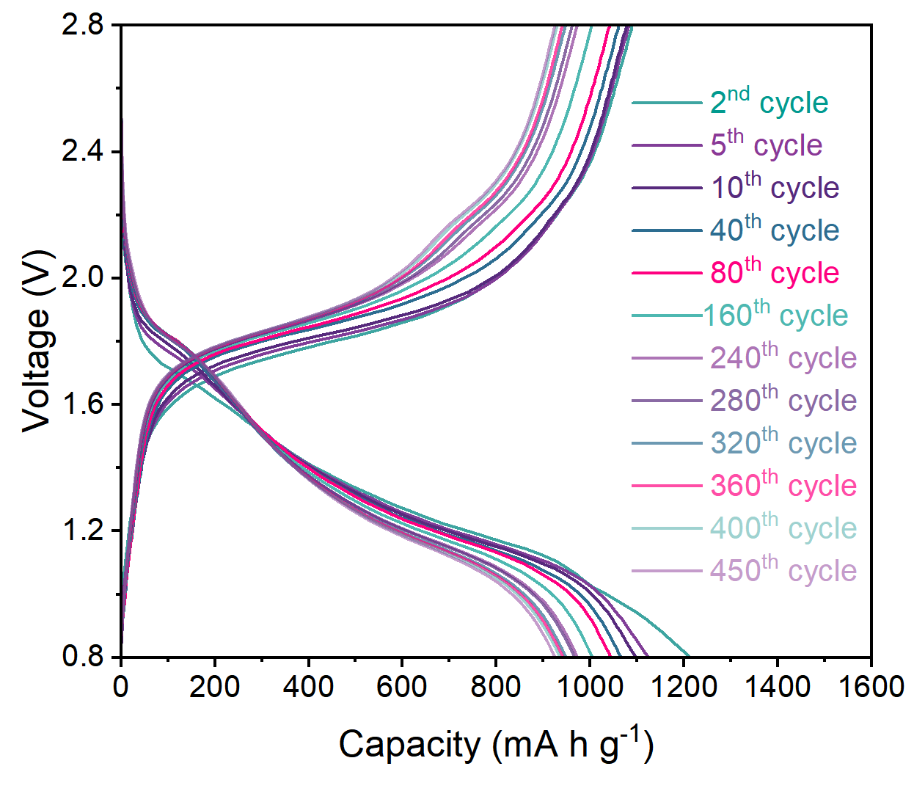
**

**Figure S15**. Discharge-charge profiles of Na-S batteries with the LHCE-1.0 at 0.2C.


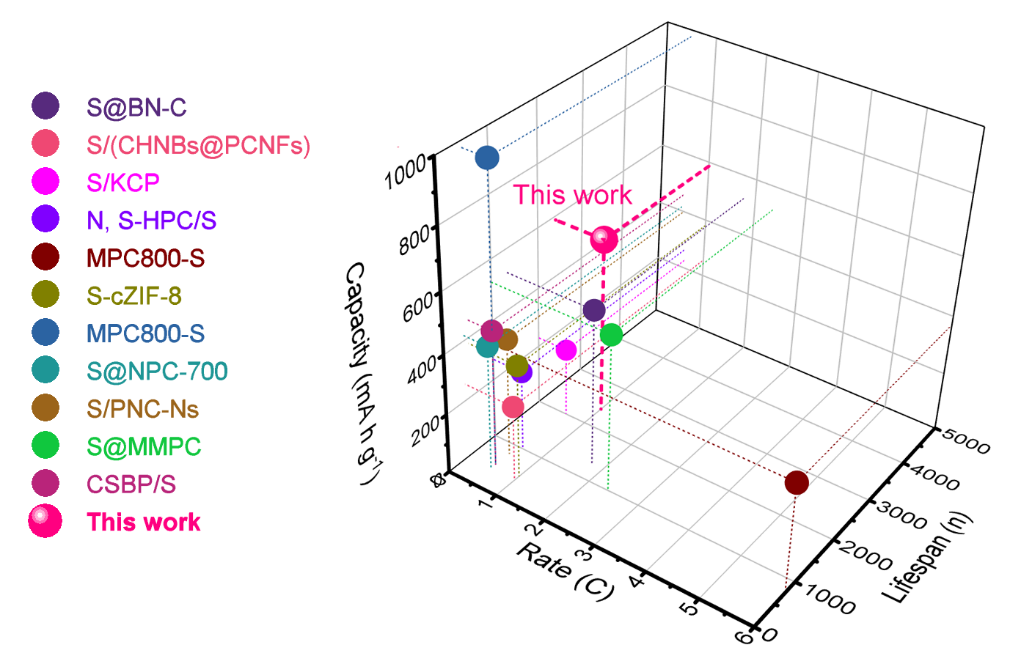


**Figure S16.** Comparison of long-term cycling performance for this work and literature.

**Figure S17.** Cycling performance of Na-S batteries with the LHCE and a high sulfur loading.

Na-S batteries can harvest a high reversible discharge capacity of 981.3 mA h g^-1^ at 0.5C and even realize a high capacity retention of 570 mA h g^-1^ after 110 cycles.


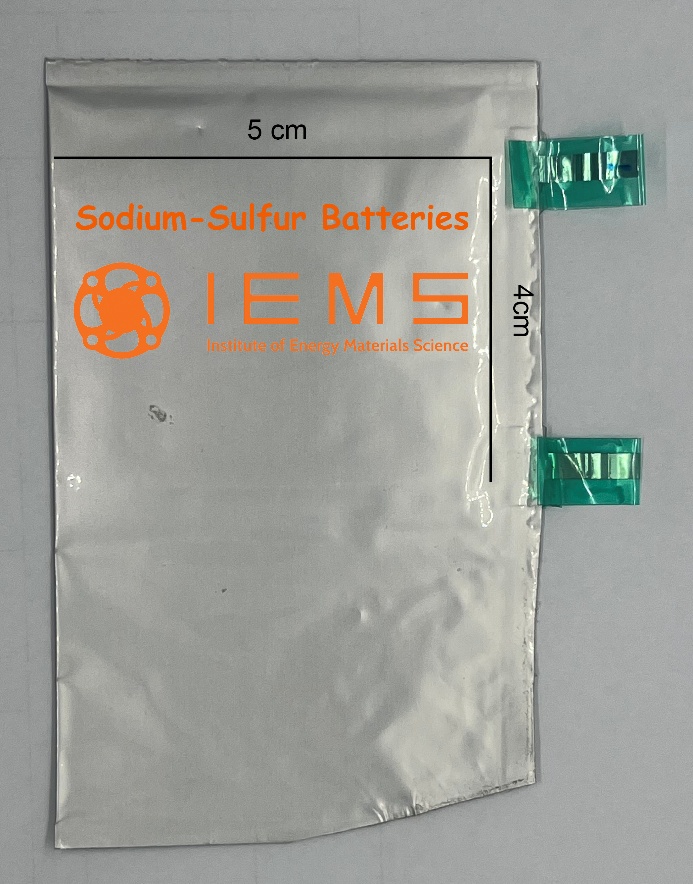


**Figure S18.** Photograph of the Na-S pouch cell designed in this work.

**Table S1.** The summary of parameters and overall performance of Na-S pouch cells reported until now.

| Cathode material | Areal loading content (mg cm^-2^) | Electrolyte | E/S ratio  (μL mg^-1^) | Sodium thickness (μm) | Current density/capacity | Energy density (W h kg^-1^) | Size  (cm×cm) | Reference |
| --- | --- | --- | --- | --- | --- | --- | --- | --- |
| ZCS@S | 5 | 1.0M NaClO4 in  DEC:EC + 5 wt % FEC | unknow | unknown | 0.1 A g^-1^  /unknown | 384 | 3×5 | [1] |
| S/P-Fe_2_O_3_@Fe-PPy | unknown | 1M NaFSI in TEGDME + 1wt% NaNO3 | unknown | unknown | 50 mA g^-1^/699 mA h g^-1^ | unknown | 5×5 | [2] |
| Ni-B@GO/PC/S | 8.9 | 1M NaTFSI in PC/FEC | unknown | unknown | 0.1 A g^-1^  /677 mA h g^-1^ | unknown | unknown | [3] |
| Na@SA Fe-N | unknown | 2 M NaTFSI in PC/ FEC | unknown | unknown | 0.2 A g^-1^  /897.6 mA h g^-1^ | 602.2 | 2×2 | [4] |
| S/NHC-InN_5_ SACs | unknown | 1 M  NaClO_4_ in PC: EC+5 wt% FEC | 4.5 | unknown | 2.0 A g^-1^  /490.7 mA h g^-1^ | unknown | 6×7 | [5] |
| S@Mn/NC | 1.8 | 1M NaFSI in TEGDME + 1wt% NaNO3 | unknown | unknown | 50 mA g^-1^/666 mA h g^-1^ | 840 | 5×6 | [6] |
| Y SAs/NC-S | 2.3 | 1 M  NaClO_4_ in PC: EC+5 wt% FEC | 4 | 100 | 0.2 A g^-1^/500 mA h g^-1^ | unknown | 6×6 | [7] |
| **Carbon/sulfur composite** | **2** | **Meso-ordering electrolyte** | **6** | **100** | **0.1C/912 mA h g^-1^** | **977.5** | **5×4** | **This work** |

**References**

[1] H. Liu, W. Pei, W.-H. Lai, Z. Yan, H. Yang, Y. Lei, Y.-X. Wang, Q. Gu, S. Zhou, S. Chou, H. K. Liu and S. X. Dou, **ACS Nano**, 2020, 14, 7259-7268.

[2] H. Zhang, B. Song, W. Zhang, B. An, L. Fu, S. Lu, Y. Cheng, Q. Chen and K. Lu, **Angewandte Chemie International Edition**, 2023, 62, e202217009

[3] B. Wang, L. Wang, B. Guo, Y. Kong, F. Wang, Z. Jing, G. Qu, M. Mamoor, D. Wang, X. He, L. Kong and L. Xu, **Advanced Materials**, 2024, 36, 2411725.

[4] R. Bai, Q. Lin, X. Li, F. Ling, H. Wang, S. Tan, L. Hu, M. Ma, X. Wu, Y. Shao, X. Rui, E. Hu, Y. Yao and Y. Yu, **Angewandte Chemie International Edition**, 2023, 62, e202218165.

[5] G. Wu, T. Liu, Z. Lao, Y. Cheng, T. Wang, J. Mao, H. Zhang, E. Liu, C. Shi, G. Zhou, C. He, W. Hu, N. Zhao, N. Wu and B. Chen, **Angewandte Chemie International Edition,** 2025, 64, e202422208.

[6] H. Zhang, M. Wang, X.-L. Huang, S. Lu, K. Lu and X. Wu, **CCS Chemistry**, 2024, 6, 2289-2304.

[7] E. Zhang, X. Hu, L. Meng, M. Qiu, J. Chen, Y. Liu, G. Liu, Z. Zhuang, X. Zheng, L. Zheng, Y. Wang, W. Tang, Z. Lu, J. Zhang, Z. Wen, D. Wang and Y. Li, **Journal of the American Chemical Society**, 2022, 144, 18995-19007.
